# Argentine Albuminuria

**Published:** 1869-01-15

**Authors:** 


### CHICAGO MEDICAL JOURNAL.

Vol. XXVI.—JANUARY 15, 1869.—No. 2.

### ORIGINAL COMMUNICATIONS.

### *Argentine Albuminuria.*

BIOLOGICAL SOCIETY OF PARIS—M. CLAUDE BERNARD, PREST.

*Observations upon certain effects of Nitrate of Silver employed as an internal remedy; by Henry Liouville, laureate, etc., etc.*

In two communications made successively to the Biological Society in June, 1868, we endeavored to complete one of the reports of a case (of which we presented the pathological specimen) of disseminated sclerosis diagnosed during life by M. Vulpian, at la Salpetriere.

In this case was discussed the demonstration establishing clearly the long duration of certain effects of the impregnation of certain organs of the economy with nitrate of silver taken internally in pill form, and of certain serious consequences of this action which had not hitherto been noticed.

It is known by experiments and clinical researches (1), of which the last are especially due to MM. Ball, Charcot

(1) Dictionnaire encyclopedique, article *Argent*, par Ball and Charcot, 1867.

and Vulpian (2) *that nitrate of silver*, or at least the product of the decomposition that this agent undergoes in the pills is absorbed into the economy, and penetrates into the circulatory torrent (3). It is moreover known that chemical investigations, skillfully conducted by M. S. Cloëz, have directly proved this absorption, by permitting the collection by analysis, in the urine secreted in certain cases of disease under treatment at la Salpêtriére, of *silver even*, under the form of small metallic grains.

The actual case which we publish, whilst confirming these principal data, will shed perhaps some new light upon this interesting question of practical physiology.

The care referred to is that of a woman of thirty-four years, J. C. B——, who, during several years, manifesting the very characteristic phenomena of disseminated sclerosis (encephalic and spinal) was admitted in 1868, to No. 14 Salle St. Mathieu, in the infirmary of la Salpêtriére, service

---

(2) Charcot et Vulpian, *Emploi du nitrate d'argent dans l'atax ie locomotive.* Bulletin de Thérapeutique, 1862.

(3) The pills of nitrate of silver are made as well with bread crumb, as with powder of marsh-mallow, or with simple syrup. It is clear that in these pharmaceutical preparations a very large portion of the nitrate of silver is modified. Some investigations made by M. S. Cloëg upon freshly prepared pills, have demonstrated that four-fifths, at least, of the salt or silver is *decomposed*, and passes into an insoluble condition, principally under the form of oxyd of silver, and of metallic silver, and perhaps of insoluble salts of an organic acid.

As to the part which remains soluble, it it not even absolutely certain that it remains in the state of nitrate.

Moreover, after that, it is averred that the nitrate of silver undergoes, even in the pills, almost complete decomposition; but, from the point of view which we occupy, we attach to this result only secondary importance.

It is certain, in fact, that the silver has been absorbed in the cases of our patients; for, as we shall explain later, *we have assured ourselves of it by analysis of the urine.*

Charcot et Vulpian, *sur l'emploi du nitrate d'argent dans l'ataxie locomotrice.* Bulletin de Thérapeutique, 1862, p. 1. After this epoch (1862) M. Cloëz had in fact succeeded in extracting a metallic globule from an analysis of urine.

of M. Vulpian, and whose autopsy we have also made during the present year. The confirmatory specimens have been presented by us to the Biological and Anatomical Society (1).

Amongst the numerous medications to which this patient had been subjected, without final success, but always with some amelioration, was *nitrate of silver*.

She had been subjected to it during six years in the following manner :

On the second day of August, 1862, they began with two pills of nitrate of silver each day.

On the eighth of August, when there was manifested a slight amelioration, she was put upon three pills.

From the eighth of August, 1862, to the twentieth of April, 1863, she pursued the treatment with three pills per day.

This was continued nine months, or two hundred and seventy days. From which we may conclude that about seven hundred pills were taken, after which they were discontinued.

The pills, made with care, contained 0.01 centigramme of nitrate of silver ; which amounted to about seven grammes, which were thus successively introduced.

Unfortunately the amelioration was temporary, and it became necessary to resort to other medicaments.

Many were employed without great success.

There was, however, no return to the nitrate of silver, and when we saw the patient upon her last appearance at the infirmary (April, 1868), she assured us that she had taken no nitrate of silver for five years. This was probably true.

We were struck by the tint, approaching to bistre, of the abdomen, and of the colored line relieved upon the edges of the gums. It should be stated that with us she had no further medication with silver in any form.

---

(1) See Bulletin de 1868, juin et juillet.

At the autopsy, we determined at once with M. Vulpian, under simple inspection, that the kidneys and the plexus choroides were considerably impregnated by the passages of the nitrate of silver.

Later, it was easy for us to establish that the supra-renal capsules also appeared to have undergone this influence.

The microscope and chemical analysis, as we shall indicate later, will complete the confirmation of all our preconceptions.

The kidneys, in fact, would exhibit after section a characteristic appearance. They were, as it were, studded, especially in the cortical substance, both superficial and intra-pyramidal, with little black, livid, isolated points presenting themselves under the form of a fine dust, with grains disseminated, but fixed, and imparting to a light and uniform touch the sensation of a beard recently shaved. These were the glomeruli of Malphigi, very apparent, stained by the metal, and appearing altogether and alone, as in the most successful anatomical preparation which we can make for their demonstration.

If prepared in the fresh state, this same appearance may be almost as perfectly recognized after four months of preservation in pure alcohol.

Under the microscope these may be positively determined to be the glomeruli of Malphigi.

It is indeed these, but these alone, which are stained. Their livid color slightly brownish, contrasting with the surrounding tissue. They were either stained in mass and uniformly, as by a blue or violet wash, or tinted in spots, and in addition, outside of the general discoloration, there were certain points, and little zones very clearly defined, of a bistre or sepia color, or nearly black. To us it was very plain in this case, that not only the surface, but even the texture of all or a portion of the glomeruli was affected; at times even the alteration seemed profound (1).

(1) This observation, confirmed moreover by another absolutely similar which we have recently made (September, 1868), in the case of a woman

None of the ducts have ever appeared to us to have undergone any great modification in their tint. In some places it seemed, indeed, that some masses were more deeply stained and tinted yellow.

But this could not be compared with the special and characteristic silvery tint of the glomeruli. On the other hand, what was more clear, and appeared to us of the greatest importance to note and to investigate with care, was the existence of numerous alterations, suggesting the granular-fatty lesions of the renal tubes affected with morbus Brightii.

Now there was in this nothing surprising, since, during life, the urine of our patient had, upon several occasions, after the exhibition of silver, yielded albumen to the tests of nitric acid and heat. It contained no sugar, but its density was 1040. Now it is interesting, I think, to compare this production of albumen and this renal lesion with the evident impregnation and the deep tinting of the glomeruli of Malphigi, consequent upon the treatment with nitrate of silver.

It is clear that there may thus be produced an *argentine albuminuria*, just as under other circumstances, almost identical, there is produced by another metal a *Saturnine albuminuria*. (*1*)

If observations of this kind should be confirmed, the practitioner should take them into serious consideration in the internal administration of silver. (2)

---

who took, during two years nitrate of silver, differs from what has been observed by the majority of authors, who have indicated up to this time that the tissue surrounding the glomeruli was alone colored, also: Frommann, Ein Fall argyria etcr., in Virchow's archives, 1859, t. xvii. Also: Ball et Charcot cite plus haut.

(1) Olivier, *De l'Albuminurie Saturnine.* Archives de Medecine, 1863.

(2) Later, in September, 1868, a new fact has likewise presented itself to our observation at la Salpetriére, but it is unfortunately incomplete as a clinical datum. A woman had, during two years, taken nitrate of silver repeatedly, in pill form, and at the autopsy we discovered plainly traces of

In every case, an examination of the urine of these patients ought always to be made carefully *before* and *during* this special treatment. It may serve to regulate it. For ourselves, we should make an absolute rule to suspend, or to diminish considerably, the metallic medication from the appearance in the urine of the smallest trace of albumen.

Before concluding what refers to the *kidney* itself, we should say that sections of the pyramids did not furnish us with so satisfactory evidences of argentine impregnation as those which resulted from the examination of the cortical substance, to which the most decided alterations seemed to be confined.

This same argentine impregnation of the *supra-renal capsules*, suspected by us when examined by the naked eye, was confirmed in a very clear manner by histological examination.

But here, also, it was in spots that the more deeply tinted points were distinguishable. There were zones of a dark ochre tint deepening to a sepia color. The cells participated in this tint, and appeared more or less intense.

The alcohol had preserved in them, after four months, this new property contracted by disease.

The tint, upon simple inspection of the plexus choroides, suggested that they also were involved. They were equally well preserved, thus colored in alcohol during four months. Their color was a deep yellow bistre, and it was likewise darker in spots; they were also blackish. Under the microscope they could be observed much better. The more deeply tinted points were perfectly well seen, like grains of sepia color.

It would have been regarded as a preparation of the plexus choroides made designedly with a solution of nitrate

---

impregnation with the metal of the kidneys (glomeruli), and the alterations of advanced morbus Brightii (granular fatty tubules); but it was impossible to recognize the results from a thorough examination of the urine during life.

We refer to this case solely from an anatomico-pathological point of view.

of silver, a reactive agent employed very successfully, as is well known, in histological investigations.

Our researches have likewise been extended to the lungs; but, although we thought that we recognized a slight tinting of the elements of this tissue—the difficulty being here increased from the possibility of confusion with the pigmentary matter so common and the condition termed carbonaceous—we will not decide as positively upon their argentine impregnation.

The other organs have not been examined by us in this connection. In no instance did they offer, upon simple inspection, anything perceptible.

We might, indeed, from the preceding limited examinations, be content to affirm the existence of the silver deposit, and its persistence in different organs of the economy. Proceeding to the examination with certain chemical reagents under the microscope, we may say that we have employed acetic acid, glycerine, alcohol, ammonia, hypochloride of lime, without inducing the least change in the color acquired by the parts tinted by the silver impregnation. But it was no longer so when we used *nitric acid*, and especially *cyanide of potassium*. Both of these, but in different different degrees—the greatest effect resulting from the cyanide of potassium—decolorized completely the glomeruli, and gave a general uniform amber-yellow tint to the preparation where previously the isolated silver tints had appeared so conspicuously. (*)

Finally, a new investigation, more decisive, perhaps, and certainly more delicate, has yet again furnished to us a new element of certainty.

From the data of M. Vulpian, M. Cloëz determined once more to attempt the detection of the silver itself.

---

(*) MM. Ball and Charcot, who had already made the same observations with reference to the majority of these reagents, have hence drawn the conclusion that this solubility of the blackish granules demonstrated that they consisted of an *albuminate of silver*, and not of a *chloride* (*Dictionnaire Encyclopedique*, article *Argent*, deja cité).

In this instance it was not from the urine, but from the kidney itself, that this distinguished chemist was enabled to extract, by the process of cupellation, a portion of the metal itself, which, at the termination of the experiment, appeared in the form of a little granule, so that we were enabled to present to the Biological Society, extracted from the half of a kidney, *a globule of metallic silver* of the size of a small pin's head, resistant, relatively heavy, almost round, and of characteristic lustre.

The experiment was thus altogether complete and perfect. We shall not insist upon the interesting consequences which may be deduced from this rare case, since we find related only the case of *Guens*, as susceptible, in some respects, of comparison. (*)

The case was that of an epileptic, who for sixteen years had discontinued the use of nitrate of silver, and at whose autopsy traces of impregnation with the metal were found in various tissues. In our case, the treatment had been discontinued but for five years.

But these new facts should be specially noted, that it was the texture of the glomeruli of Malphigi, and not *the surrounding tissue alone*, which was impregnated ; that there were present the lesions characteristic of Bright's disease, apparent in the profound alterations of glomeruli due to medication (*argentine albuminuria*) ; that the supra-renal capsules were impregnated ; and finally, that it was possible, having established the actual facts by ocular demonstration and by microscopic examination, to separate chemically, under the form of the argent itself, the incontestable proof of the physical and physiological action. Legal medicine, as well as therapeutics, may deduce from this data of the highest practical interest. W. H.

---

(*) Van Guens (Donders d'Utrecht), Archiv. fur die Hollandischen Beitrage zur Natur und Heilkunde. Berlin, 1857.

